# A toddler with an unusually severe polyarticular arthritis and a lung involvement: a case report

**DOI:** 10.1186/s12887-022-03716-1

**Published:** 2022-11-04

**Authors:** Pietro Basile, Giulia Gortani, Andrea Taddio, Serena Pastore, Federica Corona, Alessandra Tesser, Egidio Barbi, Alberto Tommasini

**Affiliations:** 1 Department of Human Pathology in Adult and Developmental Age “Gaetano Barresi”, University of Messina, Via Consolare Valeria 1, 98124 Messina, ME Italy; 2 Institute for Maternal and Child Health IRCCS “Burlo Garofolo”, 34137 Trieste, Italy; 3 Department of Medical, Surgical and Health Sciences, University of Trieste, Via Dell’Istria, 65, 34137 Trieste, Italy

**Keywords:** COPA Syndrome, Interferonopathy, Polyarticular arthritis, Interstitial pneumopathy, Case report

## Abstract

**Background:**

COPA syndrome is a rare hereditary inflammatory disease caused by mutations in the gene encoding the coatomer protein subunit alpha, causing excessive production of type I interferon.

This case is a reminder for the general paediatrician, highlighting the relevance of the association between arthritis and lung involvement in toddlers.

**Case presentation:**

We report the case of a 2-year-old girl with intermittent limping and joint pain. Her family history was relevant for a Still disease with lung involvement in the mother. Physical examination showed moderate wrist swelling. Laboratory findings on admission showed an increase in inflammatory markers, positive rheumatoid factor, antibodies antinuclear antibody (ANA) and cyclic citrullinated peptide (anti-CCP). Wrists’ ultrasound documented synovial thickening, and chest X-rays showed an unexpected severe interstitial pneumopathy. Genetic testing confirmed the diagnosis of a heterozygous mutation of the COPA gene in c.841C > T (p.R281W). Janus kinase treatment was started (baricitinib, 4 mg daily per os) with a remarkable improvement in limping and joint pain after two weeks.

**Conclusions:**

In cases of recurrent arthritis with family history and multiple involvement organs, a genetic disorder should be suspected and genetic testing should be performed. Furthermore, this case suggests that therapy with jak inhibitors may be effective and safe in interferonopathies.

## Background

COPA syndrome is a rare hereditary inflammatory disease caused by mutations in the gene encoding the coatomer protein subunit alpha, causing excessive production of type I interferon [1, 2]. These mutations are inherited as an autosomal dominant trait with a highly variable phenotype. The disease commonly begins in childhood; the most typical clinical features mimic polyarticular juvenile idiopathic arthritis (JIA), in association with diffuse alveolar haemorrhage (DAH), interstitial lung disease and nephropathy. A lung CT scan could highlight DAH signs (patchy ground-glass opacities, focal alveolar condensation), interstitial lung disease, sub-pleural or parenchymal cyst traction and signs of pulmonary fibrosis. Although COPA syndrome is a rare but well-described disease, we believe this case has an educational value for the general paediatrician, highlighting the importance of the association between arthritis and lung involvement in toddlers.

## Case presentation

A 2-year-old girl was seen for a 6-month history of intermittent limping and joint pain, previously treated with acetaminophen or nonsteroidal anti-inflammatory drugs (NSAIDs) with little benefit. The child was born preterm (27 weeks gestation), she suffered from mild bronchopulmonary dysplasia and bilateral renal dysplasia, and her mother reported a Still disease with lung involvement. In the previous months, an ultrasound of lower limbs’ joints had been performed, documenting a mild right hip effusion.

Physical examination showed moderate wrist swelling**;** objective chest evaluation, saturation values and a slit lamp eye examination were unremarkable.

Blood sampling showed average blood count, increased ESR, positive antibodies ANA and anti-CCP, hypergammaglobulinemia, hypercomplementemia and highly positive rheumatoid factor (Table 1). Kidney function was normal, as well as urine analysis, without proteinuria or haematuria**.**

**Table 1:** Laboratory findings on admission

Parameters	Results	Reference interval
White blood cell (× 10^3^/μL)	11.13	6.00 – 10.80
Neutrophils (× 10^3^/μL)	4.69	1.50 – 8.50
Lymphocytes (× 10^3^/μL)	5.11	2.00 – 8.00
Hemoglobin (g/dL)	12.6	10.5 – 13.5
Platelet (× 10^3^/μL)	296	150—450
Creatinine (mg/dL)	0.34	0.26 – 0.55
Aspartate aminotransferase (U/L)	5.01	4.33 – 7.21
Alanine aminotransferase (U/L)	44	23—46
ESR (mm/hr)	40	2—20
CRP (mg/dL)	5.1	< 5.0
IgG (mg/dL)	1364	300—1070
IgM (mg/dL)	243	50—170
IgA (mg/dL)	115	< 90
NSE (mcg/L)	56.7	< 18.0
RF (U/mL)	574	< 20
C3 (mg/dL)	144	85—142
C4 (mg/dL)	55	12—41
ANA	1:640 (positive)	
Anti-CCP (U/mL)	> 340.0	Positive > 10
ANCA	negative	
ENA	negative	
Interferon signature score	Positive (24)	< = 2.2

Abbreviations: *ESR* Erythrocyte sedimentation rate, *CRP* C-reactive protein, *NSE* Neuron specific enolase, *RF* Rheumatoid factor, *C3* Complement component 3, *C4* Complement component 4, *ANA* Anti-nuclear antibodies, *Anti-CCP* Anti-cyclic citrullinated peptides, *ANCA* Anti-neutrophil cytoplasmic autoantibody

Due to the discrepancy between the mild articular involvement and the marked increase of rheumatoid factor and acute inflammation indexes, the girl was admitted in order to perform further testing.

**Fig. 1:**
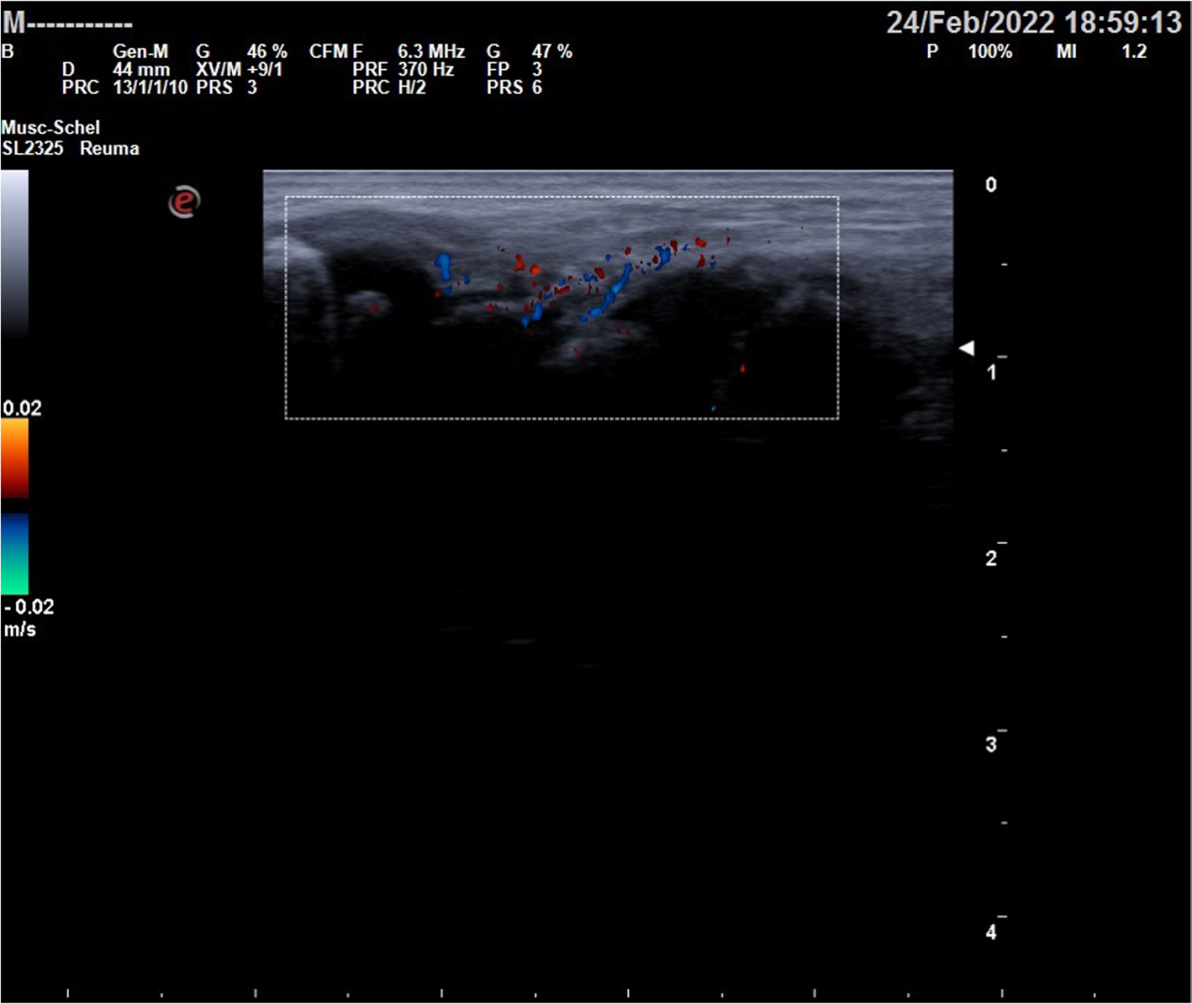
Wrists’ ultrasound shows synovial thickening with increased vascularity on colour Doppler

**Fig. 2:**
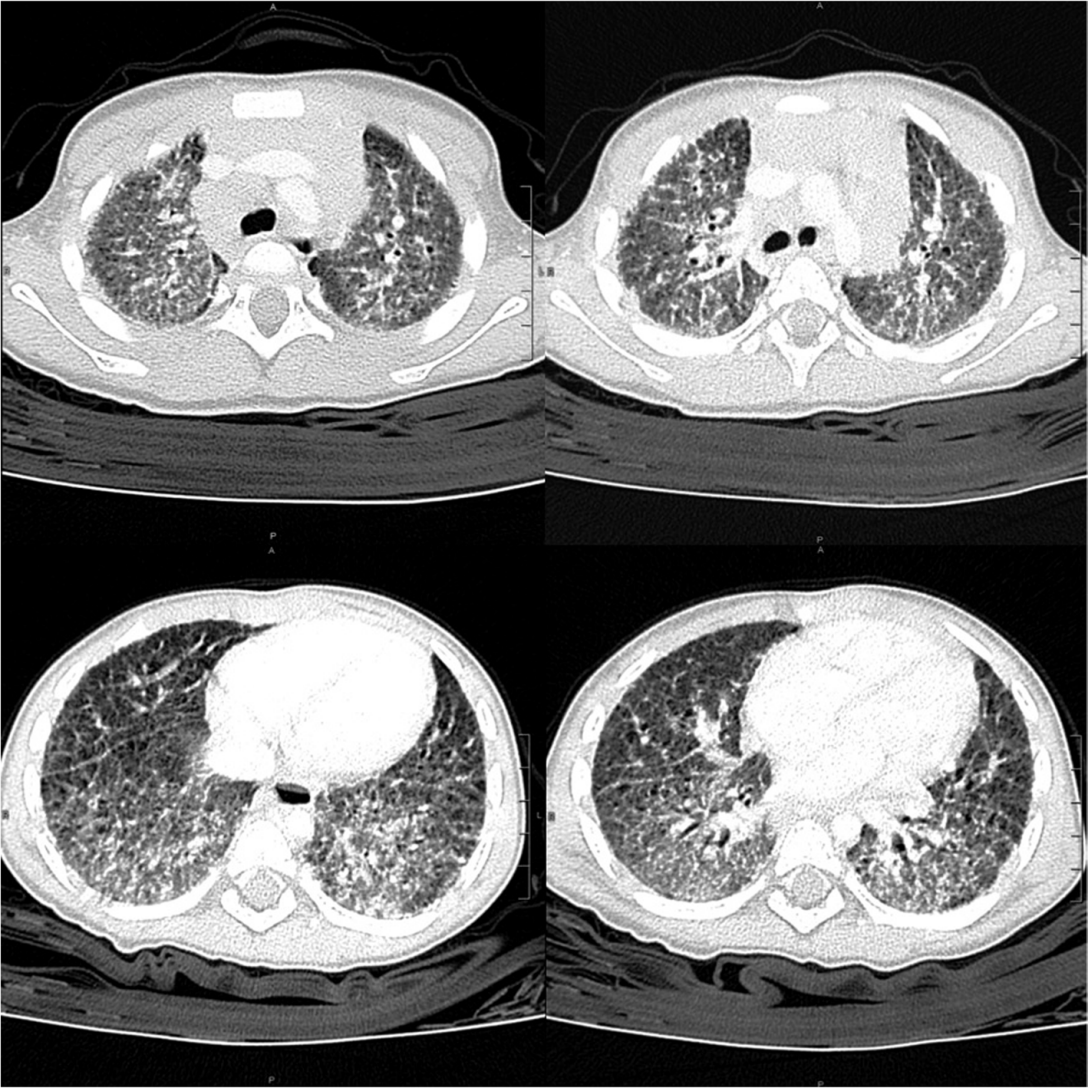
Chest CT scan shows diffuse marked thickening of the pulmonary interstitium with ground-glass areas, especially at the lower lobes. Bilaterally, numerous tiny cystic formations associated with bronchiectasis are evident at the subpleural level. Massive lymph node swelling in all mediastinal stations is present

Wrists’ US revealed a synovial thickening (Fig. 1); the hips and the abdominal US were regular. Other laboratory tests, carried out to rule out neoplasms and possible autoinflammatory pathologies, showed a mild increase of specific neuronal enolase (NSE) and a significant rise in interferon signature (Table 1). Mantoux test, quantiferon, urinary homovanillic and vanilmandelic acids were negative. Due to the increased NSE, a chest X-ray was performed to exclude the presence of neuroblastoma. The X-ray indicated a diffuse interstitial disease, non-compatible with a diagnosis of neuroblastoma or bronchopulmonary dysplasia**.** Remarkably, the toddler did not show any sign of respiratory distress. A pulmonary CT highlighted severe interstitial pneumopathy with diffuse fibrosis, patchy ground-glass opacities, cystic areas, bronchiectasis and mediastinal lymphadenopathy (Fig. 2).

The multi-system presentation, the family history, and the discrepancy between the very mild articular involvement and markedly increased rheumatoid factor prompted the suspect of a COPA syndrome. A genetic test proved a heterozygous mutation of the COPA gene in c.841C > T (p.R281W).

Janus kinase inhibitor treatment was started (baricitinib, 4 mg daily per os) with a remarkable improvement in limping and joint pain after two weeks.

## Discussion and conclusions

In children with arthritis and lung involvement various diagnoses should be considered, such as systemic lupus erythematosus, vasculitis (e.g. granulomatosis with polyangiitis), systemic juvenile idiopathic arthritis, dermatomyositis. The association of arthritis and renal involvement also evokes systemic lupus erythematosus and vasculitis.

In this case, the young age at onset of the disease and the absence of other specific clinical and laboratory features suggestive of Lupus (skin rash, kidney involvement), or dermatomyositis (Gottron sign, asthenia), prompted us to rule out a genetic inflammatory disorder.

The association of lung and/or renal involvement with arthritis should suggest a COPA syndrome among differential diagnoses. In children with COPA, pneumopathy can be initially asymptomatic and eventually progressive, with the development of alveolar bleeding, severe fibrosis and end-stage respiratory failure**.** Generally, in the presence of significant lung involvement, the clinical presentation is characterized by chronic cough, tachypnoea, and rarely haemoptysis. Lung examination may highlight retractions, crackles and signs of hypoxia such as cyanosis or clubbing.

The glomerular disease has been documented in half of the patients with COPA. The main histologic features are crescentic glomerulonephritis and focal mesangial hypercellularity with immune complex deposits, ranging from isolated IgA deposits to “full-house” immunofluorescence. Polyarticular chronic inflammatory arthritis can be severe with involvement of the cervical spine with vertebrae fusion.

In this patient, besides rheumatological symptoms, physical chest examination, saturation values and respiratory rate were standard. A chest X-ray performed to rule out other pathologies unexpectedly showed a severe picture of interstitial pneumopathy, confirmed by pulmonary CT. The genetic test confirmed COPA Syndrome’s diagnosis revealing heterozygous mutation of the COPA gene in c.841C > T (p.R281W). Disease transmission is autosomal dominant, but mutations de novo are also possible. The genetic test performed on the mother was negative while the genetic test performed on the father and siblings is still ongoing.

Remarkably, an NSE moderate increase has been found in inflammatory diseases [3] and neuroendocrine cell hyperplasia in some COPA variants [4]. We ruled out neuroblastoma for the non-typical chest X-ray appearance, the normal level of vanilmandelic and homovanillic acid in the urine and the negative abdominal US.

COPA syndrome does poorly respond to steroids or other immune-suppressive treatments such as methotrexate and TNF-alpha inhibitors. In the literature, two cases of COPA syndrome have been reported responding to Janus Kinase inhibitor, one to baricitinib and one to ruxolitinib [5]. Regarding pulmonary involvement, it is not currently possible to define the response to treatment. Larger series with long follow-ups are needed to evaluate the possible arrest of disease progression.

Janus kinase inhibitors are supposed to act by hampering Type I interferon and IL6. Although not yet approved in children in Europe, they are approved in other countries for use in other childhood inflammatory diseases, allowing to demonstrate and describe their pharmacokinetics and safety profile [6, 7]. The expected benefits are the improvement of joint symptoms and the prevention of lung lesions progression. In our case, although a re-evaluation of pulmonary CT was not yet performed, treatment with baricitinib allowed a rapid resolution of the rheumatological symptomatology, with normalization of the inflammatory markers and IFN signature in a few weeks.

The limit of this report is the short follow-up after starting treatment with baricitinib; the point of strength is the take-home message highlighting the association between arthritis, family history and lung involvement.

Paediatric practitioners should be aware of the risk of a genetic disorder in front of recurrent arthritis with family history and multiple organ involvement. Even in the absence of respiratory or renal symptoms genetic testing should be considered, being the gold standard of diagnosis.

## Data Availability

Data sharing does not apply to this article as no datasets were generated or analysed during the current study.

## Abbreviations

COPA: Coatomer protein subunit alpha
ANA: Antinuclear Antibody
anti-CCP: Cyclic citrullinated peptide
JAK: Janus akinase
JIA: Polyarticular juvenile idiopathic arthritis
DAH: Diffuse alveolar haemorrhage
ESR: Erythrocyte sedimentation rate
US: Ultrasound
CT: Computerized tomography
ACE: Angiotensin–converting enzyme
NSE: Neuron-specific enolase
PLCG2: Phospholipase C gamma 2
